# Research status and development of microbial induced calcium carbonate mineralization technology

**DOI:** 10.1371/journal.pone.0271761

**Published:** 2022-07-22

**Authors:** Jun Chen, Baolei Liu, Ming Zhong, Chuan Jing, Baoyou Guo

**Affiliations:** 1 Key Laboratory of Exploration Technologies for Oil and Gas Resources (Yangtze University), Ministry of Education, Wuhan, Hubei, China; 2 Department of Petroleum Engineering, Leak Resistance & Sealing Technology Research Department National Engineering Laboratory of Petroleum Drilling Technology, Yangtze University, Wuhan, Hubei, China; 3 Key Laboratory of Drilling and Production Engineering for Oil and Gas, Hubei Province, Wuhan, Hubei, China; University of Kurdistan Hewlêr, Kurdistan Region, IRAQ

## Abstract

In nature, biomineralization is a common phenomenon, which can be further divided into authigenic and artificially induced mineralization. In recent years, artificially induced mineralization technology has been gradually extended to major engineering fields. Therefore, by elaborating the reaction mechanism and bacteria of mineralization process, and summarized various molecular dynamics equations involved in the mineralization process, including microbial and nutrient transport equations, microbial adsorption equations, growth equations, urea hydrolysis equations, and precipitation equations. Because of the environmental adaptation stage of microorganisms in sandy soil, their reaction rate in sandy soil environment is slower than that in solution environment, the influencing factors are more different, in general, including substrate concentration, temperature, pH, particle size and grouting method. Based on the characteristics of microbial mineralization such as strong cementation ability, fast, efficient, and easy to control, there are good prospects for application in sandy soil curing, building improvement, heavy metal fixation, oil reservoir dissection, and CO_2_ capture. Finally, it is discussed and summarized the problems and future development directions on the road of commercialization of microbial induced calcium carbonate precipitation technology from laboratory to field application.

## 1 Introduction

Since the reform and opening-up, China’s economy has entered a period of rapid development. However, the industrial structure is relatively unreasonable, traditional industries dominate, and high-tech industries account for a low proportion. Economic development is frequently accompanied by the fast consumption of natural resources and the destruction of the ecological environment. Meanwhile, the shortage of energy resources forces human beings to change their thinking and develop renewable energy and various energy-saving technologies. The destruction of the natural environment is primarily induced by increased pollution, such as sand weathering, greenhouse gases, industrial waste residues, and metal pollution. A significant number of microorganisms in nature generally have a subtle impact on foundations, minerals, underground engineering, and pollutants. In traditional geotechnical engineering, people usually pay more attention to macroscopic structures, present insufficient understanding of microorganisms, and thus ignore the impact of microorganisms on the engineering field. With the realization of researchers, new green environmental protection technology has been increasingly investigated. Microbial-induced carbonate precipitation technology, as microbial mineralization technology (MICP), was discovered by Boquet [1] in the last century. In 2004, Whiffin [2] first proposed "microbe-induced calcium carbonate precipitation to produce microbial gel" and revealed that microorganisms can convert ions in the environment into solid minerals through the influence or control of organic matter. Based on this technology, Mitchell [3] designed "Bio-Earth Technology" in the field of geotechnical engineering. Since then, more and more researchers have begun to conduct in-depth research on this technology. It can be better applied to the fields such as sand control [4, 5], heavy metal fixation [6, 7], crack repair [8, 9], reservoir profile control and water plugging [10, 11], and CO_2_ capture and storage [12, 13] by changing various factors. In the future, it will be applied to a wider range of fields with the further maturity of this technology. In this paper, the microbial-induced calcium carbonate precipitation technology and related research around the world are reviewed; the influencing factors of microorganisms in different environments are summarized from the perspectives of the microbial mineralization mechanism and mineralizing fungi; its main application fields are analyzed; the current status of this technology is evaluated. The complications clarified in this stage and the future development direction will provide a certain reference for the follow-up researchers of microbial mineralization technology.

## 2 Mineralization technology

### 2.1 Mineralization mechanism

**Fig 1:**
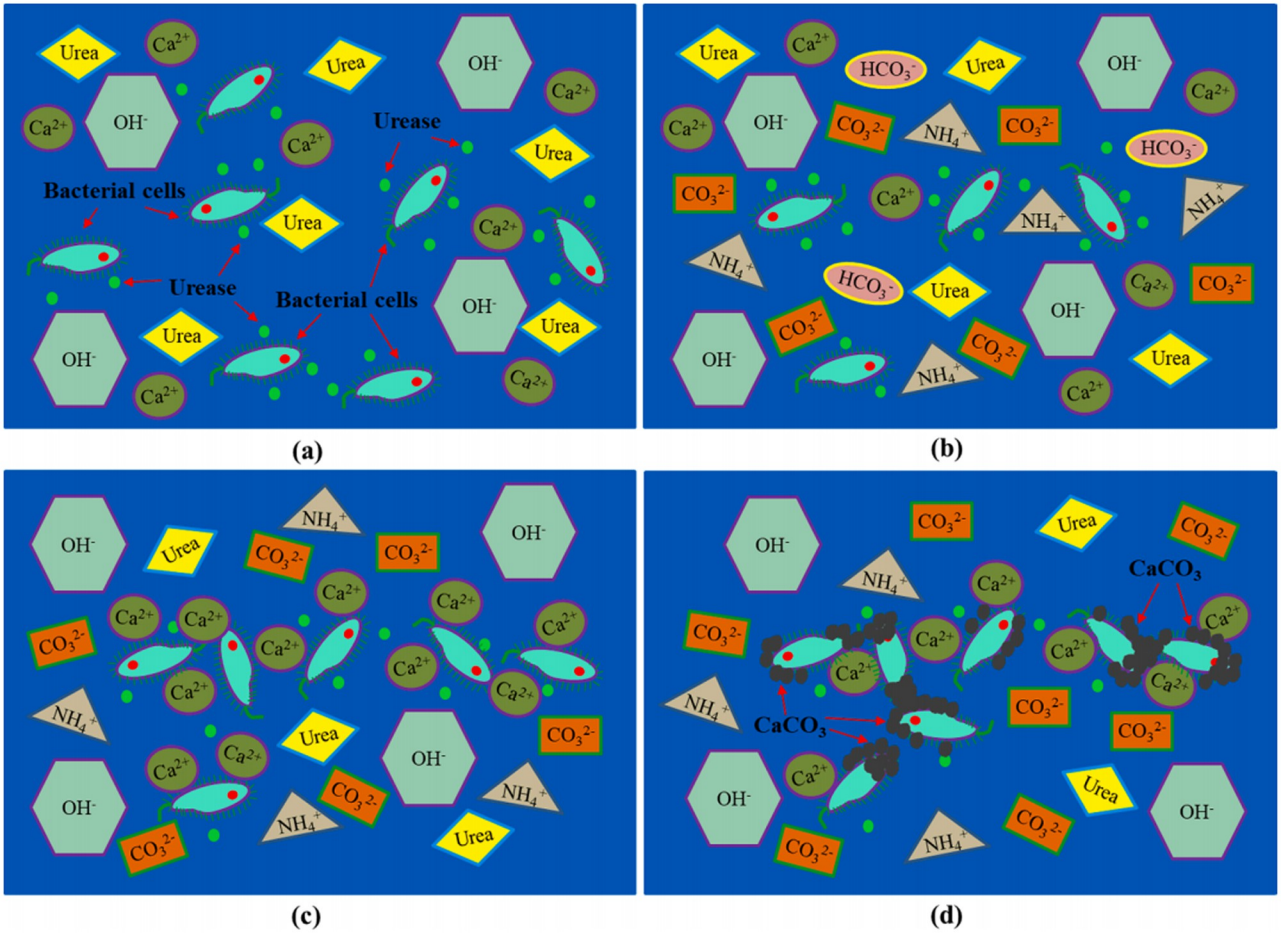
Mineralization mechanism of calcium carbonate precipitation induced by microorganism in solution. (a): Bacterial growth and urease production; (b): conversion and formation of various inorganic ions; (c): Ca^2 +^ adsorption by bacterial cells; (d): Bacterial cells as nucleation sites and deposition of CaCO_3_.

Microorganisms induce calcium carbonate precipitation including a series of chemical reactions such as biological action. It mainly utilizes a microorganism with the ability to produce urease, which can decompose urea and cause the local environmental pH to increase [14, 15], and then chemically react with the divalent metal ions in the environment or foreign. In the process of biomineralization, microorganisms not only secrete urease to hydrolyze urea but also act as a crystal nucleus for calcium carbonate precipitation [16]. Microbial cells are a negatively charged colloidal substance [17], and can drive Ca^2+^ in the solution environment to accumulate in the surrounding environment of the cell wall through adsorption, electrostatic attraction, and van der Waals force, resulting in local supersaturation [18]. The supersaturated area of the cell wall becomes the nucleation site of calcite crystals [19], contributing to the precipitation of calcite-type calcium carbonate crystals with cementation. Concurrently, the urea in the solution environment is continuously decomposed into CO_3_^2-^ and NH^4+^ under the action of urease produced by microorganisms, and the CO_3_^2-^ is transported to the cell surface and chemically reacts with the enriched Ca^2+^ to form calcite precipitation (Fig 1) [20, 21]. The main biochemical reactions during mineralization can be expressed by the following equation [22]:

$$CO{\left(NH_{2}\right)}_{2}+3H_{2}O\to 2NH_{4}^{+}+HCO_{3}^{-}+OH^{-}$$ (1)

$$HCO_{3}^{-}+H_{2}O+OH^{-}\to CO_{3}^{2-}+2H_{2}O$$ (2)

$$Ca^{2+}+Cell\to Cell-Ca^{2+}$$ (3)

$$Cell-Ca^{2+}+CO_{3}^{2-}\to Cell-CaCO_{3}^{}↓$$ (4)

Urea hydrolysis is relatively slow compared to other reactions that occur spontaneously in organisms, and urease exceeds all other enzymes in its ability to increase the reaction rate [23], with a hydrolysis efficiency of approximately 10–14 times the non-catalytic reaction rate [24]. As early as 1971, BremnerL [25] inhibited the efficiency of urease hydrolysis of urea in soil by evaluating more than 100 kinds of chemical compounds. With the discovery of the advantages of urease activity, researchers have adopted it for urea removal from yellow wine [26, 27], determination of soil enzyme activity [28, 29], disease monitoring [30–32], and microbial mineralization. Urease-producing bacteria have been valued by researchers due to their high precipitation efficiency, wide applicability, safety, greenness, and environmental friendliness of carbonates induced by urease.

### 2.2 Kinetic equations during mineralization

The MICP process involves complex biochemical reactions and hydrodynamic processes, such as microbial and nutrient transport equations, microbial adsorption equations, growth equations, urea hydrolysis kinetic equations, and precipitation reaction kinetic equations [33, 34].

#### 2.2.1 Transport equation

The majority of bioremediation techniques rely not only on the advective diffusion of chemicals to alter metabolism but also on the transport of microbial cells themselves [35]. When undertaking MICP, microbial and nutrient transport needs to be considered since this affects the location of microbial adsorption in the pores and the distribution of calcium carbonate precipitation [36]. Corapcioglu [37] builds transport equations by combining microorganisms and nutrients and constructs models based on deposition and blockage mechanisms. Chang [38] enhanced crude oil recovery under the optimization of injection parameters following the simulation results of microbial and nutrient transport in 1D core drive experiments and coupled the flow equation and continuity of microbial and nutrient transport under the conditions suitable for black oil reservoirs. Related studies suggested that the interactions between microbial flow and transport processes in multi-spatial media have a considerable impact on applications in various fields, with deeper implications for the carbon cycle and related climate change in the environment [39]. Firouzi [40] predicted the migration and deposition of bacteria in soil using single- and two-site kinetic models, discovering that the two-site kinetic model was more consistent with the observed data. Button [41] reported a strong correlation between microbial transport and nutrient concentrations. Zhao et al. [42] developed continuity equations from the Navier-Stokes equation for free flow and porous media flow, respectively, whose microbial and nutrient transport can be expressed by the following equation:

$$\nabla \cdot \vec{D}\cdot \nabla (\phi S_{W}C)-\nabla \cdot (\vec{u}C)+\phi S_{W}(\eta -k_{d})C+Q_{W}C/V=\frac{\partial \left(\phi S_{W}C\right)}{\partial t}+\phi S_{W}k_{c}C-k_{y}\rho (\sigma -\sigma_{irr}){\left(\frac{\sigma }{\phi }\right)}^{h}$$ (5)

$$\nabla \cdot {\vec{D}}_{\text{f}}\cdot \nabla (\phi S_{W}C)-\nabla \cdot (\vec{u}C_{\text{f}})-\eta \text{(}\phi S_{W}C-\rho \sigma )/Y+Q_{W}C_{\text{f}}/V=\frac{\partial (\phi S_{W}C_{\text{f}})}{\partial t}$$ (6)

Where: $\vec{D}$, Microbial diffusion coefficient, m^2^/d; *ϕ*, Porosity, %; *S*_*W*_, Water Saturation,%; *C*, Microbial Concentrations, mg/mL; *u*, Darcy Flow, m/d; *η*, Microbial growth rate, d^-1^; *ρ*, Microbial density, mg/mL; *σ*, The volume of pore media adsorbed by microorganisms; *Q*_*W*_, Daily Injection, m^3^/d; *V*, Well network control volume, m^3^; *k*_*d*_, Decay rate, d^-1^; *k*_*c*_, Blockage rate, d^-1^; *k*_*y*_, Unblockage rate, d^-1^; *Y*, Number of cells produced per unit of nutrients; ${\vec{D}}_{\text{f}}$, Diffusion coefficient of nutrients, m^2^/d; *C*_f_, Nutrient concentration, mg/mL.

The left side of Eq (5) represents microbial diffusion, migration, tropism, growth and death, and injected output, respectively; the right side refers to accumulation, blockage, and unblocking of microorganisms in solution, respectively. The left side of Eq (6) indicates the diffusion, migration, consumption, and injection output of nutrients, respectively; the right side denotes the accumulation of nutrients in the environment.

#### 2.2.2 Adsorption equation

Adsorption is an essential basis for the survival, growth, and biochemical reactions of microorganisms in a system. The microorganisms and sugars are injected into the designated sites and then are inevitably subject to adsorption loss by the porous media. Especially, the porous media composed of different minerals are non-homogeneous and have favorable and unfavorable adsorption sites for microorganisms [40]. The kinetic process of microbial adsorption can be fitted by various models, such as the primary kinetic model and the secondary kinetic model [43, 44]. It has been documented that the adsorption process is controlled by the surface of the porous medium, and its adsorption rate decreases exponentially with depth [45]. Conde et al. [46, 47] derived the Gibbs free energy change, enthalpy change, and entropy change in the adsorption process from the van Hove equation. Additionally, Su [48] argued that the Arrhenius equation can calculate the activation energy and adsorption rate during adsorption and determine the type of adsorption by thermodynamic parameters. Paassen [49] investigated the transport, precipitation, and attachment of bacteria during sand fixation with diffusion-adsorption equations. Xiao [50] characterized the bacterial diffusion mechanism during MICP by a microfluidic chip. Following existing microbial mineralization theories, researchers have mostly neglected the effect of flow rate on bacterial sorption kinetics. Therefore, Ning et al. [51, 52] considered the effect of different flow rates on microbial adsorption kinetics based on microbial adsorption kinetics in a dynamic hydrodynamic environment. The microorganisms are adsorbed from the solution onto the surface of the porous medium as a mutual result of the kinetic interaction between the flow and the microorganisms. This can be expressed in a first-order linear adsorption equation between the two phases:

$$\frac{\partial C_{\text{bs}}}{\partial \text{t}}=R_{\text{r}}-R_{\text{d}}$$ (7)

Where: *C*_bs_, Adsorption of microorganisms per pore volume in porous media; *R*_r_, Microbial retention rate; *R*_d_, Microbial desorption rate.

The microbial adsorption sites are decreasing with time. It is assumed that the equilibrium state is reached instantaneously [53], expressed as a Langmuir form dimensionless number:

$$C_{\text{ks}}=\frac{a_{k}C_{k}}{1+b_{k}C_{k}}$$ (8)

Where: *C*_ks_, Adsorption capacity per unit pore volume; *a*_*k*_, *b*_*k*_ The Adsorption Constant of a Component; *C*_*k*_, Concentration of a component in the solution environment.

#### 2.2.3 Growth equation

Throughout the history of microbial growth research, the most widely used expression for microbial growth is the Monod kinetic equation, which considers the number of microorganisms and the substrate concentration [54, 55]. Heijnen and Romein [56] suggested that microbial growth can be regarded as a coupled metabolic and catabolic process, which is irreversible and has only one substrate. In contrast, Merchuk [57] demonstrated two steps in the growth process of microorganisms: the transport of substrates from the solution to the cell surface and the transport of metabolites; their growth rate is certain. On this basis, Liu [58] argued that microbial growth involves highly complex metabolic processes, implying that the development of the Monod equation should be simplified according to various assumptions. Although microbial growth follows the Monod equation, the model exhibits nonlinearity and coupling [59]. This equation is only suitable in the exponential phase of microbial growth. The traditional Monod equation based on the Michaelis-Menten equation characterizes the relationship between the concentration of the substrate and the growth rate of the microorganism:

$$\frac{1}{C}\frac{dC}{dt}=\mu =\mu_{\text{max}}\frac{S}{K_{S}+S}$$ (9)

Where: *C*, Microbial Concentrations; *μ*_max_, Maximum specific proliferation rate of microorganisms; *K*_*S*_, Saturation constant; *S*, Substrate concentration.

Microbial growth can be constrained by not only one substrate but also dual substrates, namely, competition or non-competitive inhibition of growth, which does not exactly fit the microbial growth pattern. Therefore, many other scientists have proposed various single-substrate inhibition models or improved models based on the Monod equation, as well as growth models under specific growth conditions, in addition to the above growth kinetic equations [60, 61]. Compared with the growth kinetic equation, the kinetics of microbial metabolite production is much more complicated and cannot be obtained directly like the Monod equation. According to the relationship between the production rate of products and the production rate of microorganisms, they are classified into three types: growth-related, growth-semi-related, and growth-unrelated [62]. Roels et al. [63, 64] derived the principle of maximum carbon conservation by elemental equilibrium and applied it to microbial product growth. They revealed that the rate of product production increased with the increasing growth rate of microorganisms. Besides, the correlation between bacterial concentration and metabolites can be reflected in the study of the correlation between bacterial concentration and metabolites. In the process of establishing the microbial growth kinetic equation, it can be expressed uniformly as the following equation through the three think-related product growth equations:

$$\frac{dP}{dt}=AX+B\frac{dX}{dt}$$ (10)

Where: *A*, Output generation speed; *B* for *Y*_*P/X*_.

#### 2.2.4 Urea hydrolysis equation

According to Eqs (1)–(3), 1 mol of CO_3_^2-^ and 2 mol of NH^4+^ are produced for every 1 mol of urea consumed in the process of microbial-induced calcium carbonate precipitation hydrolysis. In the process of urea hydrolysis, the effects of urea concentration, pH, Ca^2+^ concentration, temperature, microbial growth rate, calcium carbonate precipitation, and other factors should be noted [65, 66]. There was a linear relationship between the initial urea decomposition rate and cell concentration in the earliest reports [67]. It was influenced by the control of single-cell kinetics and the maximum critical initial cell concentration [68]. Hommel [69] improved the calibration of the model under the consideration of the effects of carbonate precipitation and concentration on urea decomposition to enhance the applicability to MICP. With the urea hydrolysis characteristic curve expressing the initial rate and hysteresis in the urea hydrolysis process, Khodadadi et al. [70] concluded that the rate of urea hydrolysis is not always suitable for the evaluation of microbial urease activity, and its hydrolysis process follows the microbial growth pattern. Wijngaarden et al. [71] obtained the relevant hydrolysis equation by exploring the effect of different factors on bacterial urease activity. Through the two-dimensional model of the random distribution of particles, Van [72] derived the Michaelis-Menten equation from the amount of urea diffusion on the surface of the particles. Given the rate of the urea hydrolysis process, a convection-diffusion-reaction theory model was developed and can be characterized by the following equation [73]:

$$r=\frac{v_{\text{max}}\left[S\right]}{K_{M}+\left[S\right]}$$ (11)

Where: *r*, Urea hydrolysis rate; $\left[S\right]$, Substrate concentration; *v*_max_, Maximum response speed; *K*_*M*_, Michaelis constants.

#### 2.2.5 Precipitation equation

After the hydrolysis reaction of urea, the NH^4+^ produced will undergo a hydrolysis reaction, resulting in an increase in the ambient pH of the solution [74]. Additionally, CO_3_^2-^ reacts with Ca^2+^ in an alkaline environment to form a precipitate. Some researchers believe that the rate of calcium carbonate precipitation is more related to the hydrolysis of urea [67, 75]. Generally, the precipitation reaction of calcium carbonate is faster than the hydrolysis reaction of urea. Hence, the precipitation reaction of calcium carbonate is influenced by the hydrolysis reaction of urea. As early as last century, Lasaga et al. [76] established the basic equation for calcium carbonate precipitation. Subsequently, Noiriel et al. [77, 78] concluded that the precipitation rate of calcite was linearly correlated with saturation. Meanwhile, they assessed the microbially induced calcium carbonate precipitation rate in porous media by constructing a numerical model. Verdoes [79] can determine the precipitation rate of calcium carbonate by analyzing the expressions for both crystalline and amorphous types of precipitation. Rossum [80] derived six saturation exponential relationship equations to predict the precipitation rate of calcium carbonate in different saturated solutions. Considering the effect of transport limitations on the precipitation rate of calcium carbonate at the scale of porous media, the following equation was established with the reaction source of CO_3_^2-^ [33]:

$$r_{prec}=\left(k_{1}a^{H^{+}}+k_{2}a^{HCO_{3}}+k_{3}\right)\left(1-\frac{a^{Ca^{2+}}a^{CO_{3}^{2-}}}{k_{sp}}\right)S^{c}$$ (12)

Where: *r*_*prec*_, Calcium carbonate precipitation rate; k_1_, k_2_, k_3_, Reaction rate constant; a, Ionic activity coefficient; *k*_*sp*_, Calcium carbonate solubility product; *S*^*c*^, Effective specific area of sedimentation.

#### 2.3 Summary

MICP process is a very complex bio-chemical reaction process. It is difficult to quantify the mineralization reaction process from time and space scales. In order to simplify the model, researchers used a large number of assumptions and empirical formulas when calculated. However, unable to establish the relationship between sand permeability and strength after mineralization. Therefore, researchers need to consider multiple factors and couple multiple physical fields to model in follow-up studies.

## 3 Mineralized bacteria

Microbial mineralization is a common phenomenon in nature. More than 60 types of biominerals have been formed through direct or indirect mineralization by microorganisms, such as photosynthesis, sulfate reduction, anaerobic bacterial oxidation, and urea decomposition. Among them, calcium carbonate precipitation caused by microbial hydrolysis of urea is the most hotly researched [81–83]. Naturally occurring microorganisms capable of inducing calcium carbonate precipitation consist of cyanobacteria, sulfate-reducing bacteria, denitrifying bacteria, and the most studied urease-producing bacteria.

### 3.1 Cyanobacteria

**Fig 2:**
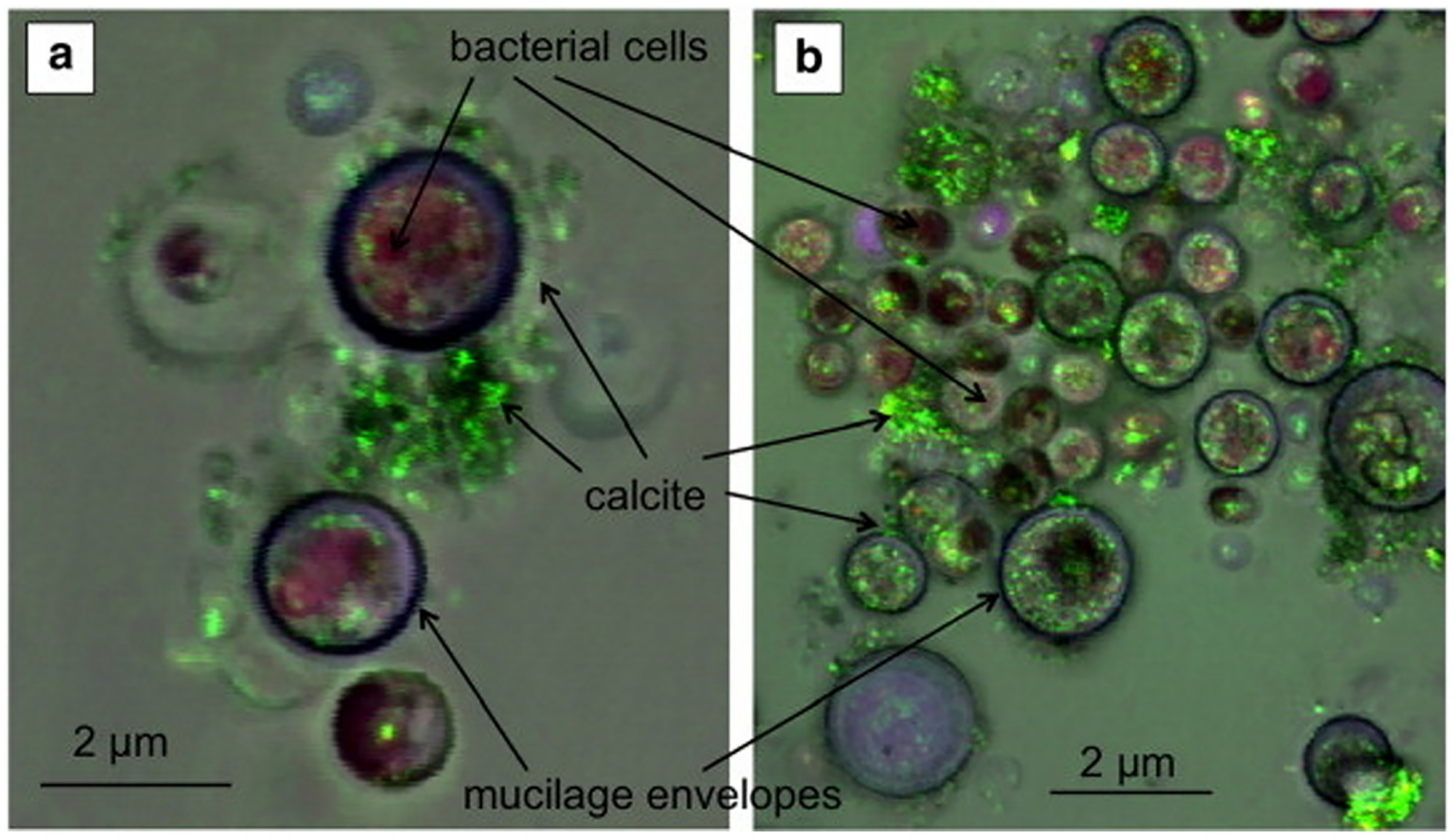
Photosynthesis during cyanobacteria mineralization.

Cyanobacteria are ancient organisms that use HCO_3_^-^ dissolved in water for photosynthesis to produce OH^-^, which reacts with HCO_3_^-^ in water to produce CO_3_^2-^. When cyanobacteria die, the extracellular polymer is degraded, and the Ca^2+^ adsorbed in the cell wall is released to form a calcium carbonate precipitate with CO_3_^2-^. The best-known examples of molecular and genetic control of microbial mineralization processes are the silica deposition by stoneflies and diatoms [84]. Furthermore, cyanobacteria will form CaCO_3_ on the cells [85]. It was reported that cyanobacteria are highly adaptable to their environment and can mineralize in not only hot springs but also nutrient-poor freshwater lakes or rivers [86–88]. In the study of the MICP process of cyanobacteria, light and UV light are critical for their MICP process, affecting the consumption of Ca^2+^ and the production of CaCO_3_. Their mineralization produces twice the number of abiotic precipitates (Fig 2) [89–91]. The main advantage of cyanobacteria over other microorganisms is that they require only CO_2_ from the environment rather than urea and carbon sources and do not produce nitrogen-based by-products, making the process less costly.

### 3.2 Sulfate-reducing bacteria

Sulfate-reducing bacteria (SRB) are widespread on Earth and play various roles, especially in anoxic land and water environments (such as soil, seawater, and riverine underground pipelines) and anaerobic extremes rich in organic matter and sulfate (such as oil and gas reservoirs, rivers and lakes, and mud). In the last century, researchers discovered that the reduction of sulfate bacteria may be responsible for the formation of different carbonate mineral compositions [92, 93]. The rate of sulfate reduction was closest to the rate of carbonate precipitation when biotic and abiotic effects on carbonate precipitation in continental shelf sediments were simulated [94]. Vasconcelos et al. [95, 96] developed a microbial dolomite model indoors to explain the role of sulfate-reducing bacteria in the precipitation of dolomite, reporting that sulfate-reducing bacteria could control the precipitation of dolomite in a low-temperature anoxic environment. Since sulfate-reducing bacteria can adapt to extreme environments, they can use organic matter on the surface of metals and other materials as a carbon source to lower sulfate to hydrogen sulfide in the absence of oxygen or very little oxygen for energy gain. Subsequently, the application of sulfate-reducing bacteria to induce calcium carbonate precipitation has been increasingly investigated in China and abroad [97, 98].

### 3.3 Denitrifying bacteria

Denitrifying bacteria are also widely distributed in nature and are found in large quantities in sewage, soil, and stable manure, where they reduce nitrate to nitrite and further reduce nitrite to ammonia and free nitrogen when soil oxygen is insufficient, leading to the increased environmental pH. The principle of microbial denitrification-induced calcium carbonate precipitation was demonstrated by Paassen et al. [99] in 2010 with fatty acid calcium salts as nucleation sites and carbon sources. Denitrification is more stable, though the rate of calcium carbonate precipitation induced by nitrate reduction may be lower than the rate of urea hydrolysis precipitation. During its reaction, calcium carbonate precipitation and microbial growth occur, while nitrogen is produced due to the reduction of nitrate bacteria. Nevertheless, calcium carbonate precipitation by denitrification is a promising technology, and its commercialization still requires further interdisciplinary research [100].

### 3.4 Urease-producing bacteria

**Fig 3:**
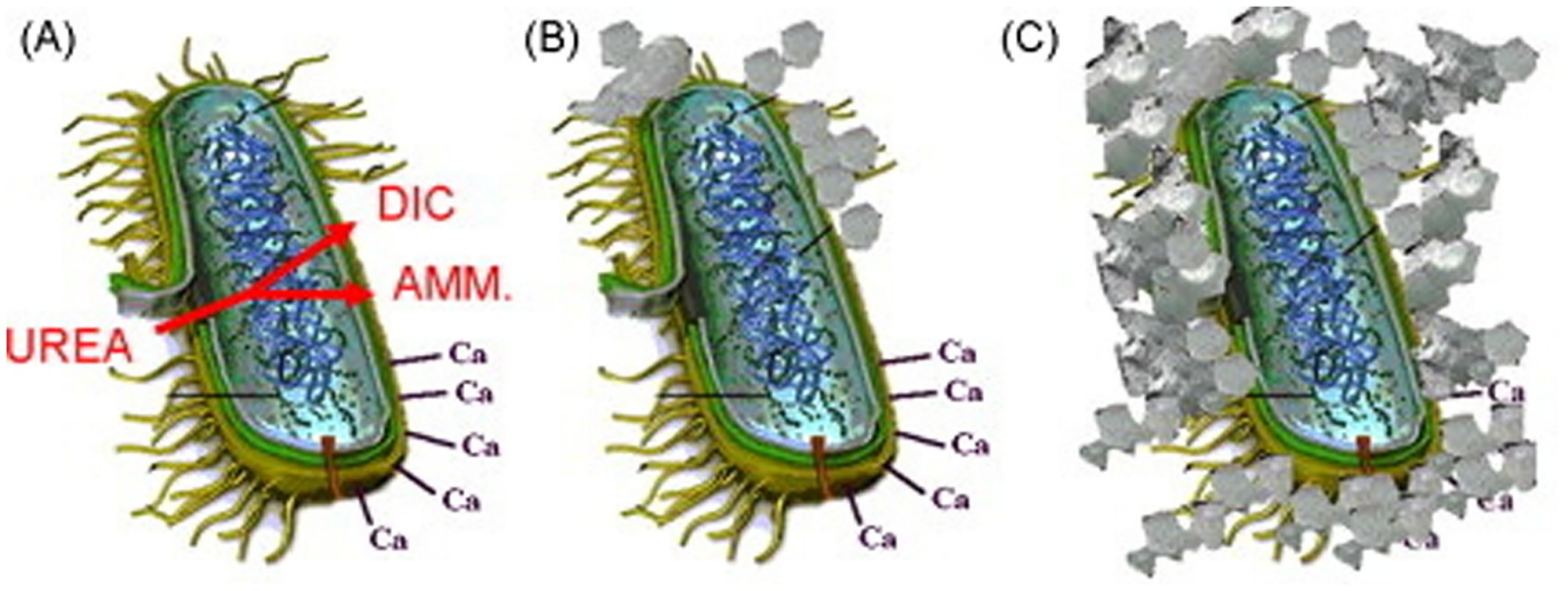
Urease-producing bacteria.

Urease is a widely occurring enzyme in nature [101], according to the most mature knowledge of this enzyme to date. Therefore, urease-producing bacteria (Pasteurella subtilis, Sporosarcina, Bacillus subtilis, and Bacillus megaterium) are known to secrete urease during metabolism to accelerate the hydrolysis of urea. Although large-scale production of highly active urease-producing bacteria is costly and affects the precipitation process [102], many researchers selected suitable media to enhance urease activity for MICP by indoor culture [24, 25, 103]. Hydrolysis of urea by urease-producing bacteria to induce calcium carbonate precipitation has more advantages than other production routes, such as simple mechanism, low cost, green and environmental protection, and the ability to produce a considerable amount of calcium carbonate precipitation in a short period of time (Fig 3) [104]. Consequently, urease-producing bacteria are most widely used in MICP.

### 3.5 Summary

There are many microorganisms that can induce calcium carbonate precipitation in nature, urease-producing bacteria were the most efficient. However, its breakdown of urea requires preferential secretion of urease, it will affect the precipitation process. Therefore, in future studies, Genetic modification and mutagenesis of urease producing bacteria as a method to improve the efficiency of calcium carbonate precipitation, Alternatively, the direct use of catalytic enzymes as an alternative to microorganisms is a simple and fast way.

## 4 Mineralization influencing factors

### 4.1 Solution environment

The growth of microorganisms in the solution environment is governed by several factors and thus impacts the effectiveness of microbial-induced calcium carbonate precipitation. The growth factors have been extensively studied. The results suggested that parameters such as temperature, pH, and substrate concentration have some influences on microbial activity [36].

#### 4.1.1 Temperature

Temperature is the most imperative factor for the growth of microorganisms, and all microorganisms in nature have their appropriate temperature range for growth. Generally, warming can accelerate the life activities of microorganisms and thus promote their growth and reproduction, while higher or lower temperatures harm the growth of microorganisms [105]. Mitchell [106] concluded that the optimal reaction temperature for most urease enzymes is 20 to 37°C. Nonetheless, Dhami et al. [107] revealed that the urease activity was still stable at 35°C; the urease activity decreased by about 47% when the temperature increased to 55°C; meanwhile, the rate of microbial decomposition of urea at high temperature decreased faster than that at low temperatures [108]. The higher the temperature, the faster the decreasing speed of the precipitation rate. As implied by the aqueous solution test, the increase in temperature caused a rapid decrease in the pH of the solution to 7.0, and the decrease in pH delayed the precipitation of calcium carbonate [109]. Moreover, the effect of temperature on microbially induced calcium carbonate precipitation was associated with the reaction time. Specifically, Ca^2+^ consumption was high at higher temperatures in the early stage, and Ca^2+^ consumption was high at lower temperatures after a period of reaction [110]. Thus, microbially induced calcium carbonate precipitation was better at lower temperatures than that at higher temperatures because the urease activity secreted by microorganisms was retained for a longer period of time at lower temperatures.

#### 4.1.2 pH

**Fig 4:**
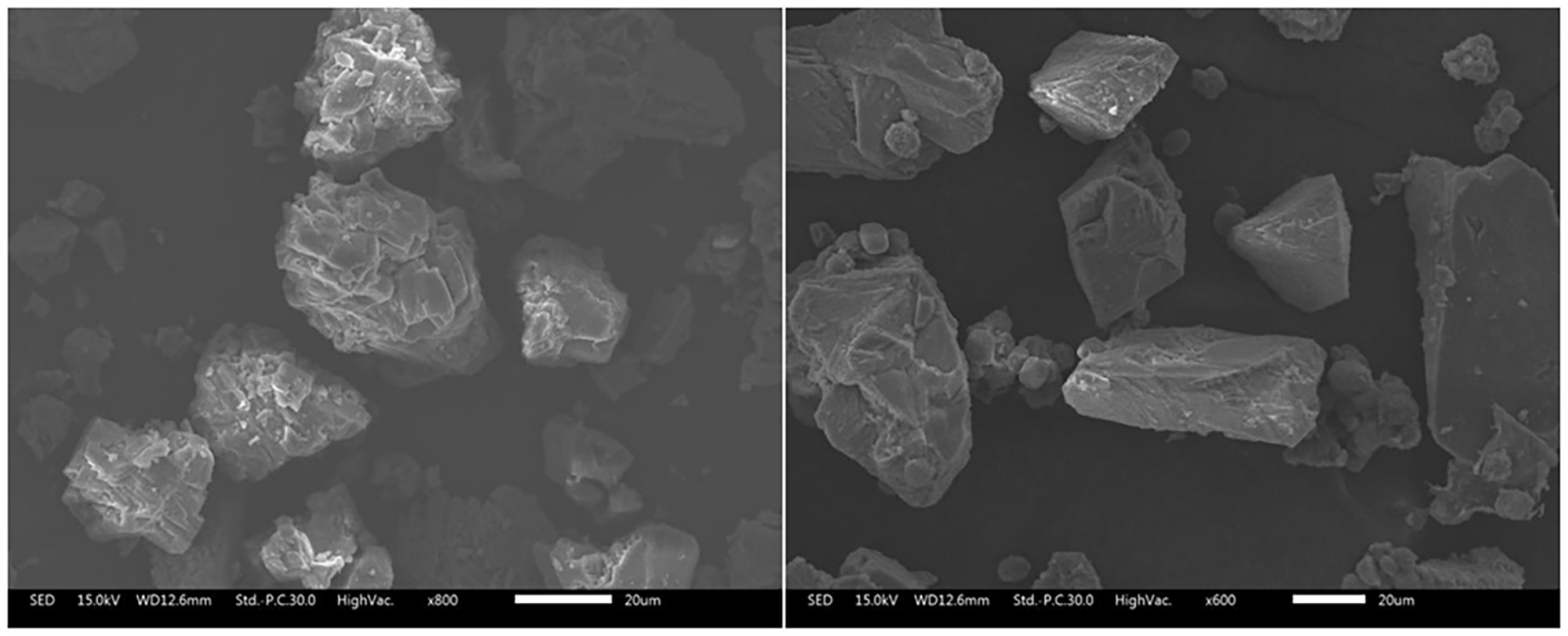
Types of precipitated calcium carbonate crystals at different pH (left 7 and right 9).

MICP occurs mostly in alkaline environments and therefore can promote the hydrolysis of urea in a specific pH range. At higher pH conditions, which are vital for the hydrolysis of urea to NH^4+^, the precipitation reaction of CaCO_3_ by microorganisms is more favorable; at lower pH conditions, the carbonate precipitation produced is dissolved. The literature suggested that the optimal pH of urease is 8 [111, 112]; the activity of urease decreases once this threshold is exceeded. However, microorganisms obtained in alkaline environments can promote their adaptation to extreme alkalinity [113]. It has good activity at pH 9 and can survive even at higher pH conditions [114]. Although the optimal pH for the induction of calcium carbonate precipitation by various microorganisms has been reported, the pH during medium precipitation is constantly changing. Therefore, Zehner [115] discovered a rapid increase in pH owing to urea hydrolysis, followed by a decrease in pH due to CaCO_3_ precipitation by monitoring the pH change in the solution environment. The higher the pH, the more unstable the calcium carbonate crystals (Fig 4).

#### 4.1.3 Substrate concentration

The MICP process requires nutrients for the growth of microorganisms and substrates for the hydrolysis of microorganisms and the precipitation of calcium carbonate to ensure that the reaction can continue. The substrate is a mixture of Ca^2+^ and urea, with urea providing the nitrogen source for microbial growth and CO_3_^2-^ for calcium carbonate precipitation. However, the concentration of the substrate has a certain impact on the effect of microbial-induced calcium carbonate precipitation. Muynck et al. [104] performed the calcium carbonate precipitation experiment, revealing that the most suitable concentrations of urea and calcium chloride were 0.5 mol/L and 0.25 mol/L. Moreover, Ca^2+^ concentration exerted a greater effect on the amount of calcium carbonate precipitation than urea concentration [116]. Theoretically, the precipitation of CaCO_3_ can be boosted by increasing the concentration of urea and Ca^2+^. Nevertheless, the high concentration of the salt solution will form a concentration difference with the cytoplasm of bacteria, and osmosis will occur, when the substrate concentration is too high (high mineralization) [117, 118]. As a result, cell dehydration and death appear, the growth and metabolism of microorganisms or the secretion of urease are influenced, and the rate of urea hydrolysis is reduced, affecting the precipitation efficiency.

#### 4.1.4 Others

There are many more factors impacting microbially induced calcium carbonate precipitation than these three. When calcium carbonate crystals are formed, microorganisms act as nucleation sites, and their concentration can significantly affect the crystal morphology and calcium carbonate yield [2, 119]. The concentration of microbial cells is the main influencing factor when the concentration of urea and Ca^2+^ reaches a certain level [120]. Meanwhile, there is a relationship between the quality of the calcium carbonate produced and the oxygen content in the environment [121]. Xu [122] changed the crystal shape by adding magnesium ions to promote the precipitation of sphalerite and inhibit the growth of calcite. Moreover, the morphology of carbonate precipitates is related to the ratio of calcium and magnesium in the solution. The main reason for the difference in morphology is that the presence of magnesium changes the saturation of the solution. With the increase of reaction time, the increase of saturation affects the precipitation. phase (amorphous phase), which subsequently crystallizes into other phases (vanadite, calcite, etc.) [123]. By studying the mineralogy of calcium carbonate precipitation induced by different divalent metal ions on microorganisms, Kim was found that different metal ions can induce the formation of carbonates with different crystal morphologies and sizes at low concentrations (e.g., calcite, aragonite, vaterite, monohydrocalcite, and calcium-strontianite) [124]. Moosazadeh [125] enhanced the precipitation of calcium carbonate by adding Fe_3_O_4_. Li and Cang et al. [126, 127] discovered that voltage strengthened urease activity and achieved increased calcium carbonate yield. The quality the final calcium carbonate produced significantly varies with the calcium source [81, 128, 129]. And different calcium sources induce crystals of different shapes, among which the calcium carbonate crystals induced by calcium chloride are the most stable [130–132].

### 4.2 Sandy soil environment

Compared with the microbial mineralization process in the solution environment, it adds a process of reactants entering the sandy soil porous medium in the sandy soil environment, so as to the effect of calcium carbonate precipitation. Their reaction rate in the sandy soil pore space will be slower than that in the solution environment ascribed to an environmental adaptation stage for microorganisms to enter the sandy soil [133]. Additionally, the mineralization effect is impacted by parameters such as sand particle size, cement concentration, maintenance temperature, and grouting method.

#### 4.2.1 Particle size

Microbially induced calcium carbonate crystals generally range in size from a few microns to tens of microns, while calcium carbonate crystals can also form agglomerates [134]. Considering that the size is hundreds of microns or more, a too-small particle size will restrict microbial growth and calcium carbonate production. Specifically, calcium carbonate of a too-large sand size tends to adhere to the surface of individual sand particles and cannot provide effective cementation for the huge sand particles, nor can it fill the pore space between the particles, resulting in the restriction of the ultimate curing effect [135, 136]. Song et al. [137] concluded that when the sand size is too small, the pore volume is small, and the calcium carbonate produced by cementation at the injection end will prevent the subsequent injection of the bacteria and weakened the overall compressive strength. Therefore, the overall strength of the cured specimen increases with the increase in the sand particle size within a certain particle size range [138]. Similarly, Mortensen et al. [139] suggested that the particle size distribution and relative density of the sand soil determine the effect of mineralization. They tested the shear velocity of the specimens and revealed that the cementation strength of well-graded coarse-grained sand was higher than that in poorly graded fine-grained sand. Owing to the large particle size span of poorly graded sands, a weak structural surface is created, leading to a lower mineralized strength. In other words, the shear strength of sands after MICP is positively correlated with the relative density [140, 141].

#### 4.2.2 Cementation concentration

Unlike the substrate concentration in the solution environment, the cementation effect of microbially induced calcium carbonate precipitation in sandy soils depends largely on the concentration of the cementation [22]. The concentration of the cementation affects not only the type, shape, and size of the crystals in the calcium carbonate deposited mineral material but also the distribution of bacteria and crystalline minerals in the pore space [142], as well as the amount of calcium carbonate generation, Deposition efficiency, pore size, and compressive strength of cemented specimens. Clarà found in fine sand, The crystal size of the calcium carbonate precipitate increased with the cement concentration, indicating that the crystals changed during the precipitation. By increasing calcium concentrations, Xu had found the crystal morphology changed from hexahedron to oblique polyhedron to ellipsoid and the Ca^2+^ ion concentration mainly affects calcium carbonate crystal morphology and size [143]. When the concentration of the cement (urea and calcium ions) was 10–250 mM, the calcium carbonate crystal size increased dramatically; when the concentration exceeded 10–250 mM, the crystal size growth became saturated [144]. Qabany et al. conducted MICP cementation of quartz sand and found that in the concentration range of 0.25 to 1.00 mol/L, the higher the concentration of the cementing solution, the larger the size and the more uneven distribution of the calcite crystals produced [36]. The calcium carbonate formed by low-concentration cementing solution is majorly adsorbed on the surface of sand particles and between pores; high-concentration cementation can form more calcium carbonate, solidify and cement sand particles, and increase the strength of specimens [145]. Qabany [146] concluded that the magnitude of the increase depended on the concentration of the cementation, though the strength of the tested samples increased after the mineralization treatment. Tang et al. [147] reported that the best consolidation effect and the best utilization rate were achieved after 3 hours of injection at a rate of 2 ml/min at a cementation concentration of 0.5 mol/L. The shear strength of the cured sand increased by 69% after 48 h of treatment [148]. Since microorganisms are limited in their ability to decompose urea under specified conditions, the concentration of cementation in mineralization experiments is generally not too high. This does not continue to improve the strength of the specimen as too-high concentration cementation inhibits the growth of microorganisms in the sand. Moreover, the calcium carbonate precipitation rate becomes slower, resulting in the lower strength of the specimen. In other words, the presence of high ion concentration weakens the mineralization and curing effect.

#### 4.2.3 Maintenance temperature

Maintenance temperature is crucial in the application of microbial sand fixation. Calcite precipitated at different maintenance temperatures has different shapes and contents, and its ability to cement the sand column significantly differs [149]. As the maintenance temperature rises, the microbial growth accelerates, and the calcium carbonate content between sand particles increases, contributing to cementing more sand and increasing the compressive strength. When the maintenance temperature exceeds 50°C, the microbial growth is inhibited, the cementing ability is weakened, and the compressive strength is reduced [108, 150]. Although more calcium carbonate is produced at high maintenance temperature, its particle size is small, and it covers the surface of sandy soil and cannot cement the particles. At lower temperatures, less calcium carbonate is precipitated, while the particles are larger, allowing it to cement the sandy soil particles and increase the strength of the mineralized sample. Related research demonstrated that the mass of calcium carbonate in the cemented sample at 50°C is three times that of 25°C, and its compressive strength is only 60% of that at 25°C [151]. Sun et al. [152] domesticated the microorganisms and increased the concentration of urea in a low-temperature environment to make them grow and multiply faster and produce more precipitation and a better curing effect in the low-temperature sand fixation test. Additionally, the temperature affects the crystal size of calcium carbonate precipitation, and the optimal temperature for curing sandy soil is 20°C~30°C [153]. At the optimum maintenance temperature, the large size of calcium carbonate crystals can effectively fill the pores of quartz sand, beneficial to improving the strength of cured samples.

#### 4.2.4 Grouting method

**Fig 5:**
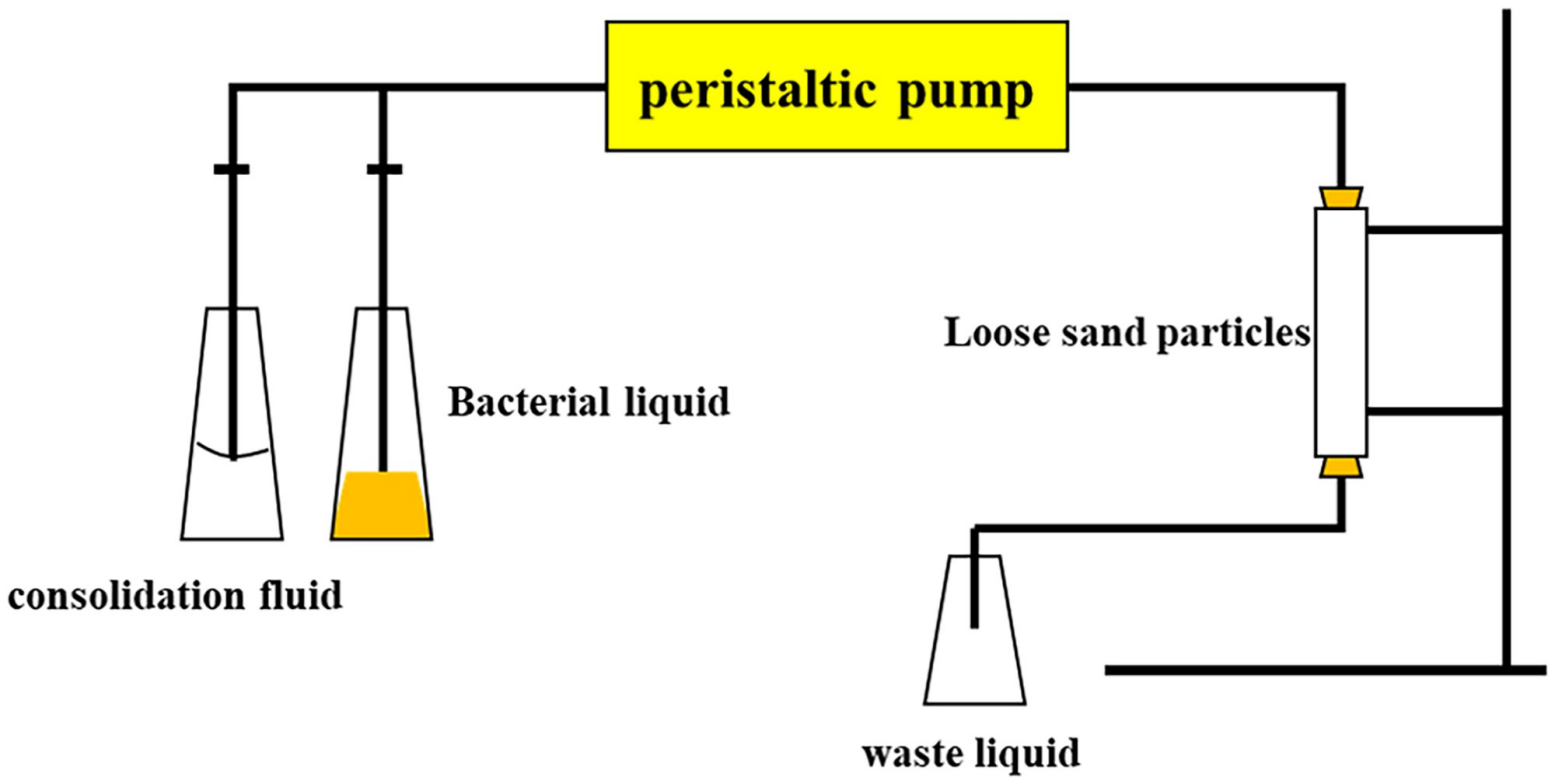
Injection method.

**Fig 6:**
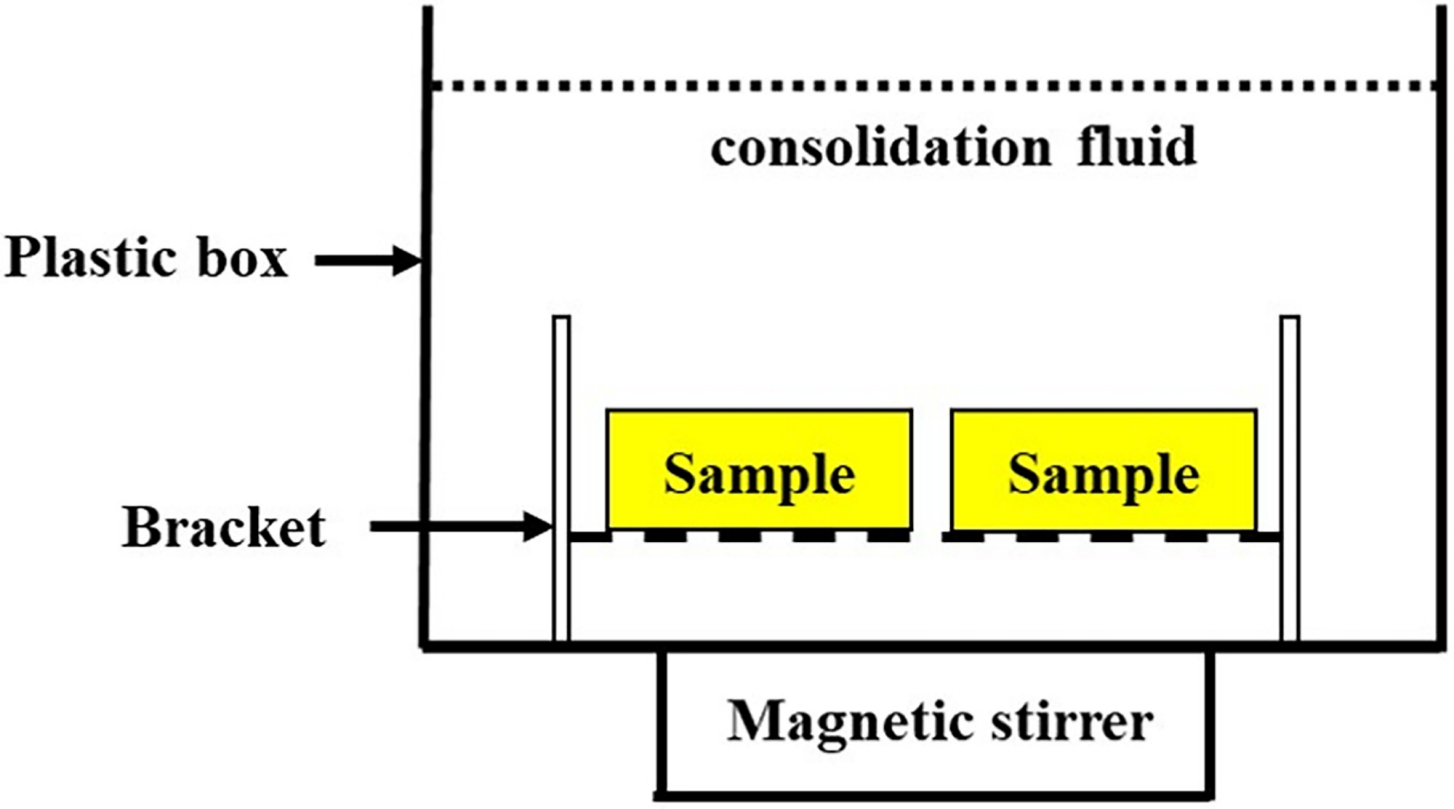
Immersion method.

There are many factors affecting the effect of microbial mineralization on sandy soil, and the research in this field remains in the exploratory stage. Thus, no mature way to solidify sandy soil is available, and the common methods contain injection, immersion, and spraying. Most of the researchers injected the cementing solution into the sand with a peristaltic pump or syringe to conduct experiments (Fig 5) [2, 154, 155]. Nevertheless, the pores of the sandy soil at the injection end are easily blocked by the generated calcium carbonate precipitation [156]. This becomes the main problem of this method. The farther away from the injection end, the less calcium carbonate is generated, and the poorer the cementation strength. Moreover, the use of peristaltic pumps and syringes will flush the microorganisms and substrate in the pore space, influencing the cementation effect. Based on this phenomenon, Wen et al. [157, 158] conducted mineralization experiments with the immersion method, in which the samples were completely immersed in a bacterial or nutrient solution, and calcium carbonate precipitates were gradually produced by natural permeation of the liquid (Fig 6). The spraying method indicates that the solution is applied to the soil surface by simply spraying the fungal solution or nutrient solution, and the solution eventually penetrates into the soil by gravity. Cheng et al. [159] applied the spray method to a 2 m long coarse sand column, discovering that the curing reaction depth was within 1 m, and the compressive strength was 850–2067 kP. The injection method can favor the production of rhombic calcite crystals if the mineralogy was considered.

#### 4.2.5 Others

These factors are not the only ones limiting the mineralization of microorganisms in the sandy soil environment. Gong demonstrated that the dry density, unconfined compressive strength, and the amount of calcium carbonate precipitation of the specimens increased with the number of injections of the cementing solution, while the curing effect did not significantly change after nine times [160]. Niu et al. [161] revealed that the strength effect of the specimens first increased and then decreased as the injection time interval increased, and 8 h was the turning point of the trend change. In contrast, Kawasaki et al. [162] suggested that the specimens cured optimally at 14 days with a 1-day injection interval can satisfy the performance requirements of the actual project. Achal presented that calcium chloride produced more precipitation by adding different calcium sources such as calcium chloride, calcium oxide, calcium acetate, and calcium nitrate to the nutritional broth after the addition of urea solution [128]. Tang et al. [163] unveiled that by adding graphene oxide to the mineralization process, calcium carbonate crystals were precipitated faster and larger in size with unchanged morphology, and the compressive strength of the consolidated sand was significantly improved. With the investigation of mineralized sand column experiments, increasing researchers have achieved the incorporation of foreign substances to enhance the mineralization capacity of microorganisms.

### 4.3 Microscopic analysis of solution and sandy soil environment

**Fig 7:**
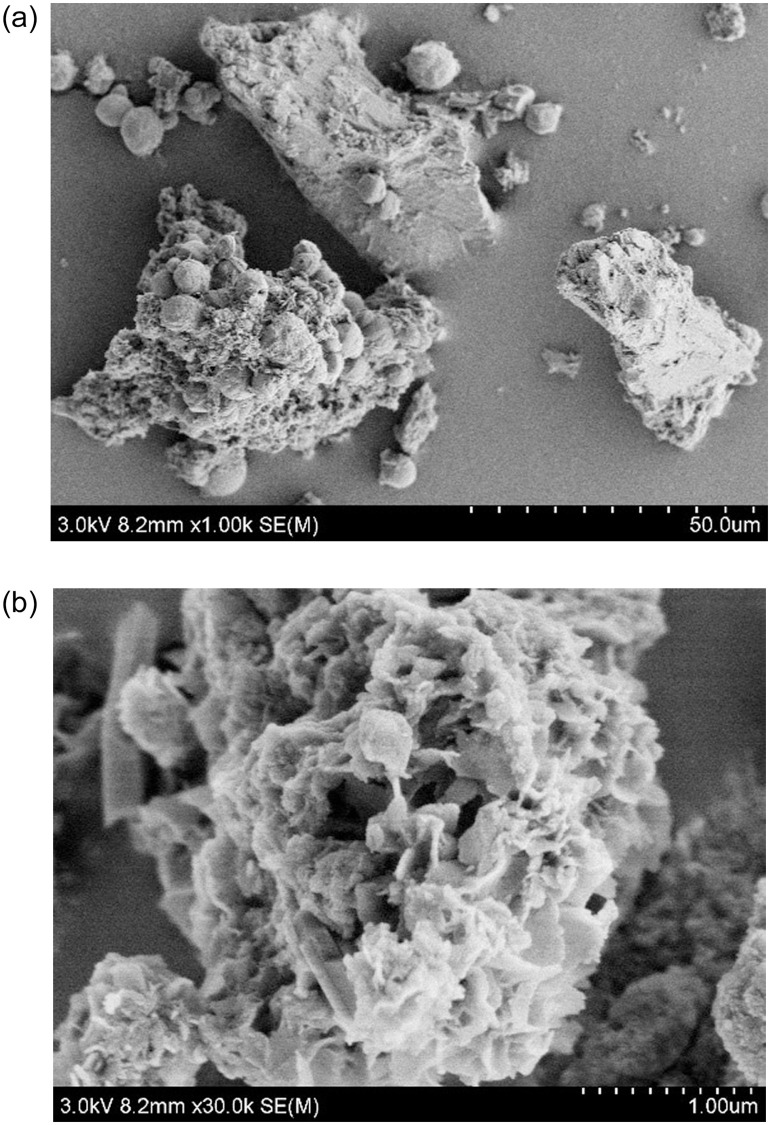
Morphology of induced calcite in shake flask solution. (a): SEM at 50.0um;(b): SEM at 1.0 um.

**Fig 8:**
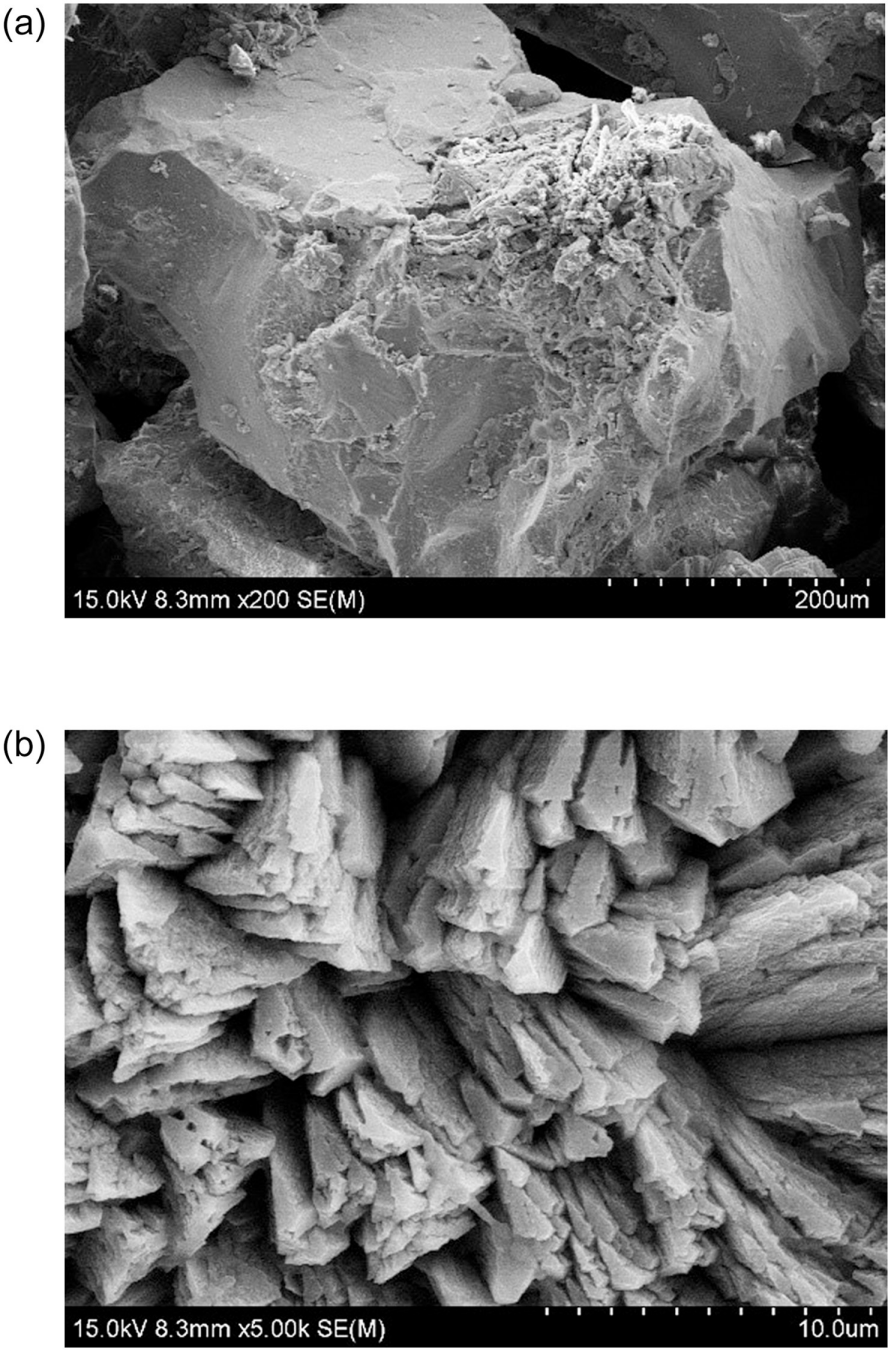
Morphology of quartz sand cemented calcite. (a): SEM at 200.0um;(b): SEM at 10.0 um.

Microbially induced calcite production in shake flasks (Fig 7) and colloidal calcite-induced mineralization in quartz sand structures (Fig 8) is exhibited in below. The SEM morphologies at different magnifications were different. It can be easily observed that the morphologies of the two were different, though the main component was calcite. The calcite induced in the shake flask experiment was mostly spherical or spherical aggregates, with a few rhombic-shaped developments. Moreover, a small number of bacterial bodies were wrapped by calcite but not completely colloidized. Besides, the calcite crystallization induced in the quartz sand environment was significantly better, mainly demonstrating oblique hexagonal lattice development, in accordance with the crystallographic morphology of calcite. Additionally, the calcite was adsorbed around the quartz sand and tightly wrapped on the surface of quartz sand.

The calcite crystals induced by the microbial solution are polycrystalline structures composed of many spherical grains interspersed with intercrystalline gaps, because the essence of calcite crystal formation induced by microorganisms is the process of biomineralization. The nucleation and growth of calcite crystals in the biomineralization process are influenced by not only the same factors as conventional crystal nucleation and growth but also various other complex regulatory factors [164]. Under the control of organic macromolecules produced by microorganisms, calcite crystals can change their growth process and morphology, resulting in multi-level growth and mutual aggregation. As a result, the interfacial energy of calcite nucleation is reduced, and the final formation of biomineralization is different from chemical methods. Bibi believed that these organic substances or bioproteins can be used as templates to induce inorganic minerals to bind to organic substrates secreted on the surface of organisms, in which they act as nucleation sites and adsorb inorganic minerals into clusters [165].

Zehner et al. [166] concluded that amorphous calcium carbonate (ACC) precipitated in the presence of initial calcite and exerted an effect on the consolidated MICP material. During the curing experiment, the microorganisms changed the chemical environment around the quartz sand surface [167]. Simultaneously, a stable biofilm was formed on the surface of quartz sand, and the environment in the pores gradually became alkaline during the biological reaction. However, the extracellular polymer of microorganisms with negative charge continuously adsorbed Ca^2+^ in the environment. Thus, calcite crystals were formed on the surface of sand particles sporadically. As the biochemical reaction process continued, more calcite crystals were formed on the surface of sand particles. With the increasing number and volume of crystals, the adjacent calcite crystals were in contact with each other and cemented into a whole, and a covering shell layer was formed on the surface of sand particles. Wang et al. [168] summarized that as the distance of calcite crystals between adjacent sand particles decreased until the two sand particles came into contact, the contact area increased further, the structural strength of the connection was enhanced, the quartz sand particles were closely intertwined, and the loose gravel was cemented into a whole. Hence, the calcite crystals formed between the grains play an essential role in the structural strength. Through its cementing effect, it becomes the skeleton structure of the gravel, contributing to the improvement of the bearing capacity of quartz sand and the bonding force between the grains.

### 4.4 Summary

Most researchers use the concentration of urea solution and calcium ion concentration as the overall concentration of the cementation for design optimization. Nonetheless, the concentration of calcium ions in the cementation and the concentration of urea in the process of microbial mineralization affect each other. Thus, both the concentrations of calcium ion and urea impact the effect of microbial sand fixation. The assessment of the concentration on its curing effect should be considered separately [169]. And most researchers only consider the strength, stiffness and permeability of the solidified sand column or soil after mineralization, durability studies on samples are still clearly inadequate.

## 5 Mineralization application areas

### 5.1 Sand control

**Fig 9:**
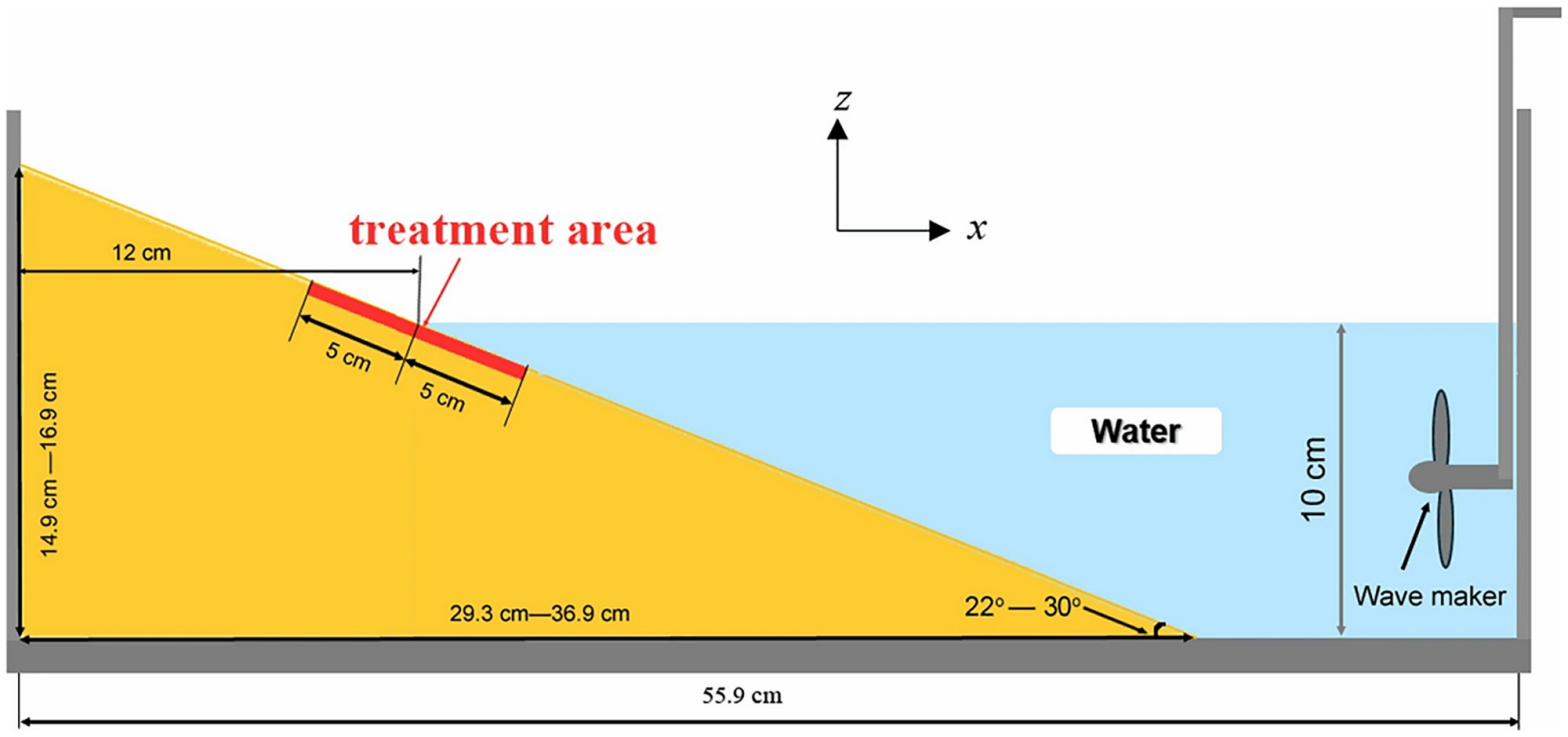
Coastal dune erosion test.

Wind erosion, coastal erosion, and desert intrusion can further aggravate the ecological damage in national environments. By spraying microorganisms and cementing solution into the wind-sand soil, which is cemented into a sufficiently strong, dense, and wind-erosion resistant monolith, Tian demonstrated that with the increase in microbial spraying frequency and colloid concentration, the density of treated sand slightly increased, and the wind erosion rate significantly decreased [170–172]. Li accelerated sand fixation and mitigated desertification through revegetation and ecological restoration by combining microbial mineralization with SCB technology [173]. Gao and Zhang et al. [174, 175] induced microbial growth by soybean extract and added Mg to the medium for desertification control in a desert area of northwest China, so as to further benefit ecosystem reconstruction. On the basis of frequent seawater inundation and erosion of coastal dunes ascribed to extreme weather and accelerated sea-level rise, researchers have treated wave-attacked dunes with microbial and enzyme-induced calcium carbonate precipitation, discovering that the mineralization effect can be effective in reducing dune erosion. However, the mineralization effect gradually decreased when the dune slope was steep, the wave intensity was high, and the wave impact time was long, and the effect was attributed to the spatial distribution pattern of calcium carbonate precipitation (Fig 9) [176, 177]. Liu et al. [178] increased erosion resistance of dykes by strengthening the surface of the dykes through mineralization techniques. With the MICP technology, the compressive strength and internal structure of loess foundation and wind sand were improved to protect the soil and water environment.

### 5.2 Architectural improvements

**Fig 10:**
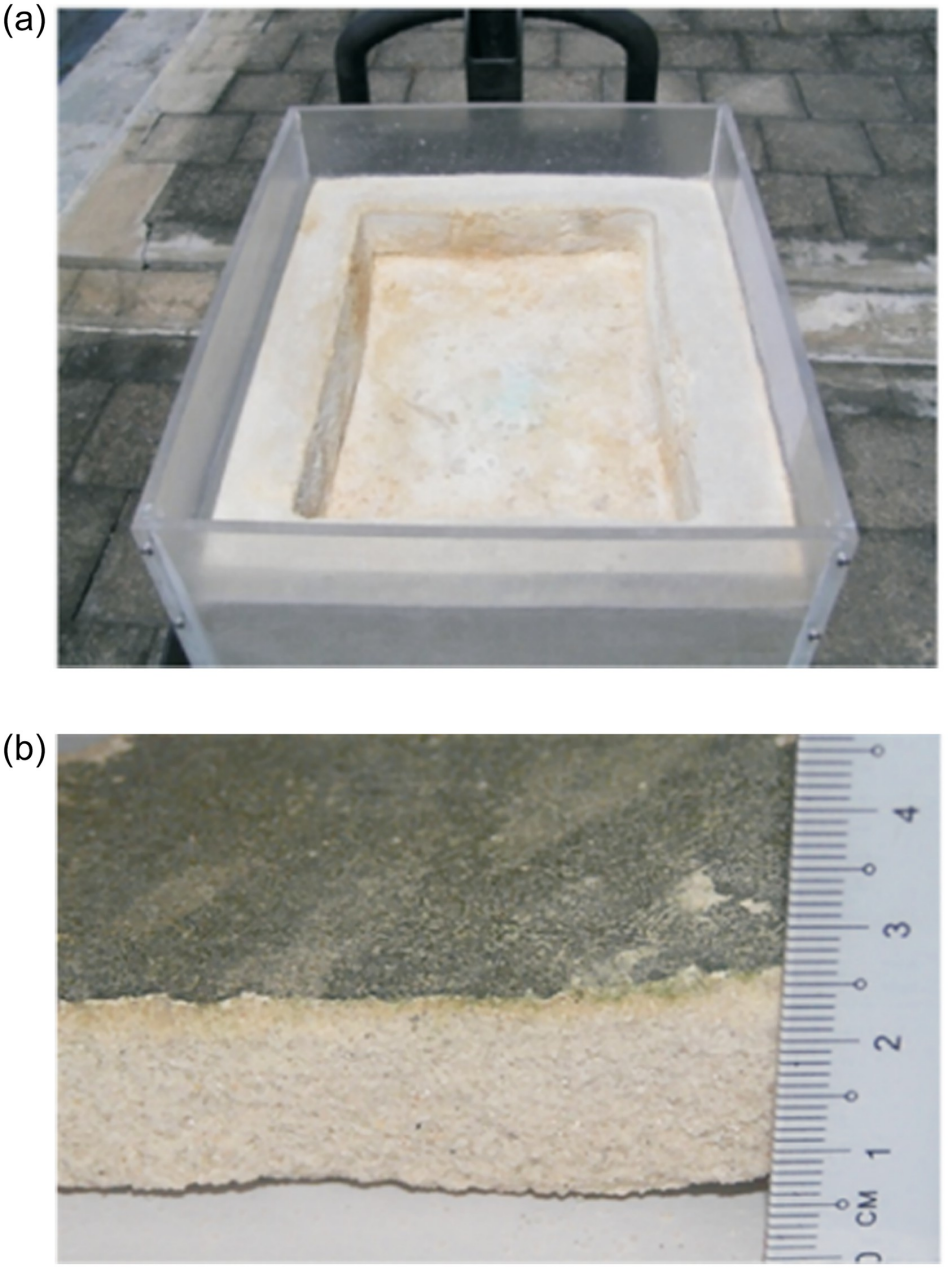
MICP application pool construction. (a): after treatment;(b) cross-sectional view of the bottom of the pond after cultivation of algae.

Since the mention of microbial cement, many researchers in the field of civil engineering have explored this technology, which has great potential for improving the performance of cement materials, restoring ancient buildings, and sealing concrete cracks. Microbial cement is considered the new era in the construction industry [179]. Unlike conventional cement products, the addition of microorganisms and substrates can reduce the water absorption capacity and boost the compressive strength and service life [180, 181]. Kavazanjian et al. [13, 182] avoided causing settlement by applying it to building foundations and repairing poor foundation soils. Wiktor weakened the permeability of the parking lot by adding microorganisms and substrates to the cracks [183]. Perito applied the technique to the monumental site of the Church of Angra [184]. Both results presented the potential of the technique. Van Paassen et al. [185] reported an experimental study of in-situ sand base reinforcement with a scale of 100m^3^ to effectively improve the bearing capacity and stiffness of the sand base. Montoya et al. [186] applied microbial grouting technology to the reinforcement of liquefied sandy soil foundations and effectively enhanced the liquefaction resistance of liquefied sandy soil foundations. Chu et al. [187, 188] utilized the MICP process to treat sand layers to create a high-strength, thin, and impermeable shell that can be used, for example, to construct ponds (Fig 10). Additionally, some of the ancient buildings in China have experienced hundreds or even thousands of years and have cracks, low mechanical properties, and low safety performance. Tsinghua University and other universities have artificially rehabilitated these cultural heritages by grouting and infiltration methods.

### 5.3 Heavy metal fixation

**Fig 11:**
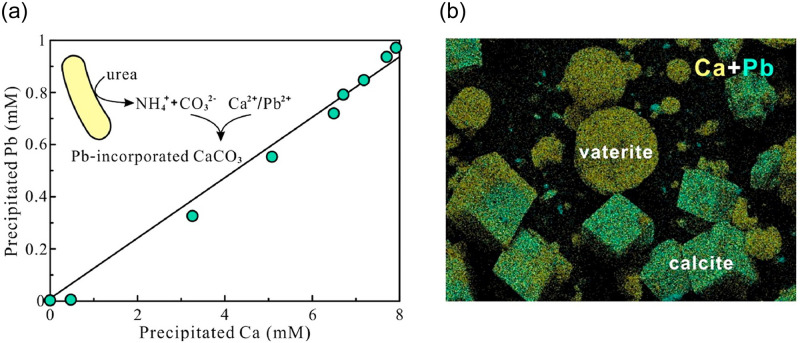
MICP application with metal Pb fixation. (a): the linear relationship between precipitated Ca and precipitated Pb; (b): Elemental maps showing the distribution of Pb.

Heavy metal pollution in the underground environment is a severe environmental problem, and the microbially induced calcium carbonate precipitation technology can make heavy metal ions precipitate as carbonates, such as Cu^2+^, Cd^2+^, Zn^2+^, Pb^2+^, and Fe^2+^, and other divalent metal ions replace Ca^2+^ in the mineralization reaction [124]. The shape, crystallinity, and precipitation rate of carbonate precipitates formed by mineralization of metal ions remarkably vary depending on their type. The toxicity of heavy metals can change the activity of microorganisms. Mugwar et al. [189] investigated those microorganisms can remove Cd^2+^ and Zn^2+^ better by adding urea in solutions with different concentrations of metal ions. Li screened six strains of metal-tolerant microorganisms, and the precipitation rate of metal at pH 8–9 was over 88% [190]. Fujita added strontium to the microbial mineralization process and revealed that strontium existed in calcite as a solid solution [191]. The rate of strontium precipitation is determined by the rate of calcite precipitation. Specifically, the faster the calcite precipitation, the more the absorbed strontium. Moghal et al. [192] performed solidification experiments on cadmium (Cd), nickel (Ni), and lead (Pb) in the soil by enzyme-induced calcium carbonate precipitation technique. They verified that the technique can retain heavy metals in the soil and reduce their mobility, and the order of desorption of the three metals by the soil was: Cd>Ni>Pb. Hui et al. [193] found with the increasing solution salinity progressively, reduced the removal efficiency of Pb, but the removal efficiency could be still as high as 89% (Fig 11).

### 5.4 Reservoir dissection

**Fig 12:**
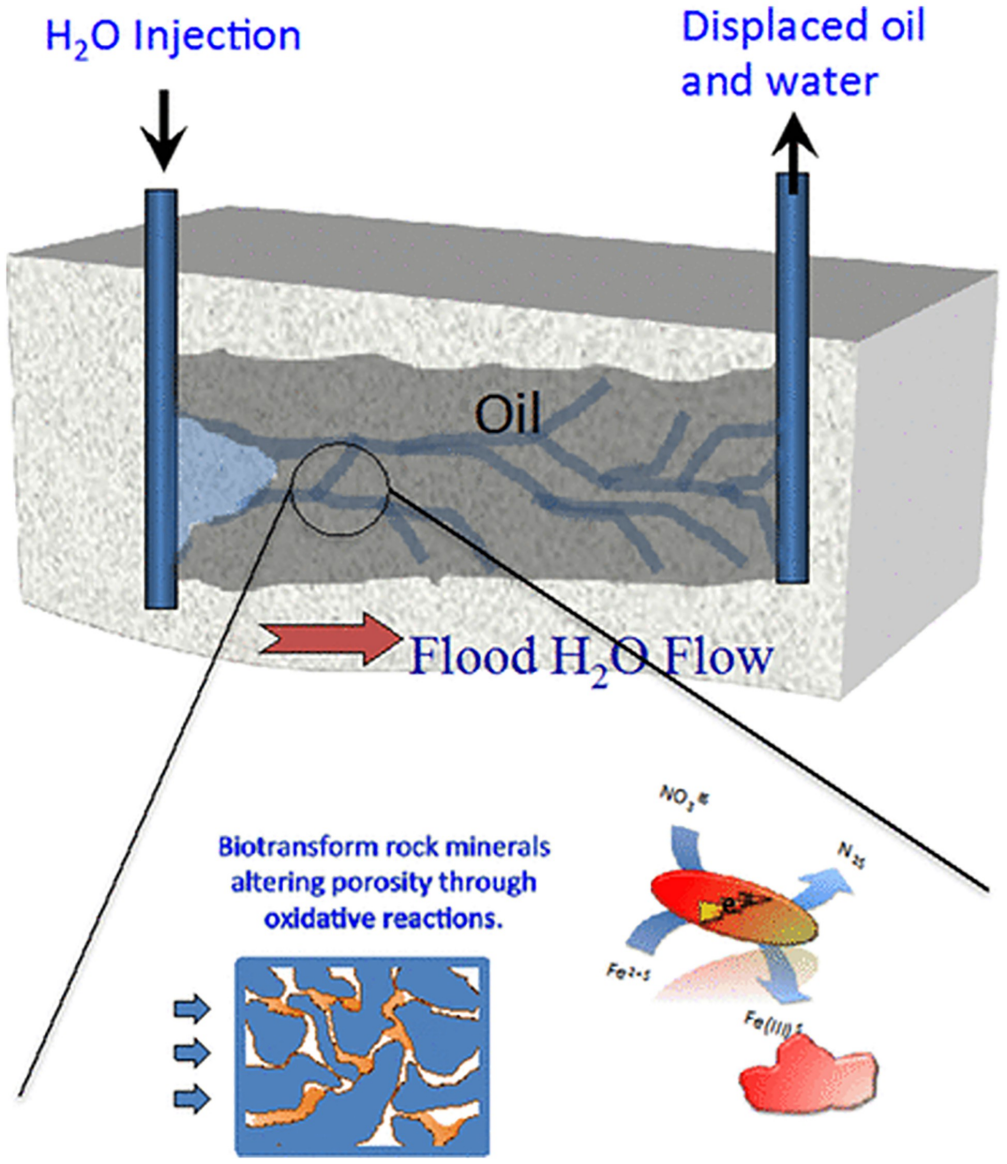
Reservoir profile control principle.

Research on oil recovery by microorganisms has been relatively mature, and researchers in the United Kingdom, the United States, Canada, Romania, the former East Germany, the former Soviet Union, Australia, and other countries have conducted excessive theoretical studies, indoor experiments, and minefield tests. Nevertheless, it is easy to form a water-driven dominant seepage channel in the late stage of oil field development due to the large difference in permeability of the reservoir matrix. Hence, the ineffective circulation caused by low water volume wave coefficient is a major problem in oilfield production. At present, there is little research on reservoir dissection and water plugging by microbially induced calcium carbonate precipitation. Nemati conducted mineralization of porous media permeability profiles with microorganisms screened in Canadian oil fields [194]. Repeated injection of colloidal fluid increased the degree of plugging of porous media and decreased permeability by 13%. Larsen improved the plugging agent based on the enzymatic calcium carbonate precipitation technique to seal natural fractures or artificial fractures in the chalk reservoir of the oil reservoir, so as to improve the field recovery [195]. Wu and Wang et al. [196] simulated the inhomogeneous reservoir indoors and used the mineralization technique. Their results suggested that the permeability decreased from 16D to 0.6D, and the crude oil recovery increased from 44% to 83%. Zhu changed the reservoir pore throat by inducing nitrate mineralization through iron-reducing bacteria, blocking the dominant seepage channel, and improving the volume wave efficiency and crude oil recovery (Fig 12) [197]. Zhong et al. [11] delayed the microbial hydrolysis of urea through the addition of glucose to the cementing solution, enabling the microorganisms to mineralize and plug the reservoir at a deeper depth to achieve enhanced recovery. Song et al. [198] investigated nitrogen-cycling bacterial-induced carbonate precipitation (MICP) to fill the pore space of porous media as a potential microbial plugging agent, contributing to improving the ripple efficiency of high permeability reservoirs.

### 5.5 CO_2_ capture

Carbon neutralization is a new concept that has been introduced in China in recent years and has attracted a lot of attention from researchers. Different from traditional photosynthesis, which converts CO_2_ into carbonate crystals that are stable and environmentally friendly, microbially induced calcium carbonate precipitation is employed to capture and bury CO_2_ for weakening the greenhouse effect. By exploring the ability of anaerobic sulfate-reducing bacteria to induce carbonate precipitation under atmospheric and CO_2_ conditions, Paul confirmed that 53% of the carbon in the precipitate was derived from CO_2_ based on carbon isotopes, and this technology converted gas to solid fixation and could sequester CO_2_ in the subsurface [199, 200]. Alshalif et al. [201] adopted carbonic anhydrase and CO_2_ in concrete based on the mineralization process. In this way, the amount of CO_2_ in the air was lowered, and the strength of the concrete was enhanced. Phillips et al. [202] sealed and strengthened the fractures in the formation with a biofilm-induced calcium carbonate precipitation technique to mitigate the possibility of CO_2_ leakage. After sealing the fractures, the cores were able to withstand three times more pressure than before sealing. The effectiveness of microorganisms on CO_2_ sequestration was researched by Okyay et al [203]. in an unnamed cave in Texas, USA. It was concluded that the rate and concentration of CO_2_ sequestration depended on the microorganism species, and the increase in pH increased the CO_2_ sequestration rate up to 78.6%.

### 5.6 Summary

In MICP studies, when CaCO_3_ is generated, high concentrations of NH_4_Cl are also generated, it can affect the atmosphere and the underground environment. How to limit the spillover of NH_3_ is a direction worthy of research, for example, consider the joint action of microorganisms that feed on nitrogen sources. There is still a problem in the application and field use of MICP technology is large-scale cultivation of bacteria, need to consider economic and time costs. Therefore, In-situ activation for bacteria in native, it is cheaper and more convenient than adding exogenous bacteria, and it is beneficial to the protection of the local environment.

## 6 Discussion and technology prospects

Microbial-induced calcium carbonate precipitation technology, as a new type of cross-process with good environmental adaptability, has developed rapidly in the past two decades due to its high efficiency, low cost, and easy control. It can quickly generate cementitious calcium carbonate to improve the internal pores and mechanical properties of soil and has little impact on the in-situ environment, presenting great application potential and high research value in the field of modern geotechnical engineering. In this paper, the mineralization mechanism of microorganisms and the microbial molecular dynamics equations involved in the mineralization process are reviewed to provide a certain reference for subsequent scholars’ theoretical research. Besides, the types of bacteria that can be used in the mineralization process are summarized. The influencing factors and microscopic differences in solution environment and sandy soil environment, as well as the main application fields of mineralization technology, lay a good foundation for further application research in the later stage.

Although this technology has achieved some achievements after years of research, it has some limitations. Compared with other methods, the process of the microbial method is slower. Most of the current experiments are indoor experiments, and there are few actual field applications. Additionally, the transition from the laboratory to the field is a challenge, the cost is high, and the by-products are harmful to the environment. The bacteria used are all urease-producing bacteria in an aerobic environment. The molecular dynamics equation in the mineralization process adopts excessive assumptions and empirical formulas, hindering the accurate evaluation of the metabolism and substrate consumption during the growth of microorganisms. Therefore, further investigation should be conducted to enable the microbial-induced calcium carbonate precipitation technology to be applied to the site on a large scale. Many strains can adapt to extreme environments such as high temperature, high pressure, high salinity, and anaerobicity. The influence of various constraints in the mineralization process on molecular dynamics should be considered in future research.
